# Ultrafast deposition of polydopamine for high-performance fiber-reinforced high-temperature ceramic composites

**DOI:** 10.1038/s41598-022-24971-3

**Published:** 2022-11-28

**Authors:** Yingjun Liu, Cheng Su, Yufei Zu, Xiaopeng Chen, Jianjun Sha, Jixiang Dai

**Affiliations:** 1 Key Laboratory of Advanced Technology for Aerospace Vehicles of Liaoning Province, Dalian University of Technology, Dalian, 116024 People’s Republic of China; 2 State Key Lab. of Structural Analysis for Industrial Equipment, Dalian University of Technology, Dalian, 116024 People’s Republic of China

**Keywords:** Structural materials, Aerospace engineering

## Abstract

The low deposition time efficiency and small thickness limit the expansion of polydopamine (PDA) application to fiber-reinforced high-temperature ceramic composites. In this work, the electric field-assisted polymerization (EFAP) route was developed to improve the deposition time efficiency of PDA coating and overcome the thickness limitation. Carbonized polydopamine (C-PDA) coating was used as the interphase of carbon fiber-reinforced ZrB_2_-based composites (C_f_/ZrB_2_-based composite) to bond rigid fibers and brittle ceramics, where C-PDA coating was prepared by the carbonization of PDA coating. Firstly, uniform and dense PDA coatings were deposited on carbon fibers (C_f_) by EFAP. The thickness of PDA coating reached the micron level (over 1800 nm) for the first time. Benefiting from the EFAP route promoting the oxidation process of dopamine (DA) and accelerating the aggregation and in-situ polymerization of DA and its derivatives on the surface of C_f_, the deposition rate of PDA coating reached 5589 nm/h, which was 3 orders of magnitude higher than that of the traditional self-polymerization process. By adjusting the EFAP parameters (e.g. DA-concentration, current, and deposition time), the thickness of PDA coating could be conveniently designed from nano-scale to micro-scale. Then, PDA coating was pyrolyzed to obtain C-PDA coating. C-PDA coating was well bonded on C_f_ without visible cross-sticking among neighboring fibers. C-PDA coating presented a layered structure and the thickness of C-PDA coating could be designed by controlling the thickness of PDA. C-PDA coating was used as the interfacial phase of the C_f_/ZrB_2_-based composite, which ensured that the composite possessed good load-bearing capacity and thermal stability. Moreover, extraordinary damage resistance of the composite was achieved, with work of fracture up to 9936 ± 548 J/m^2^ at room temperature and 19,082 ± 3458 J/m^2^ at 1800 °C. The current work provides a high time efficiency processing route for depositing PDA coating on carbon fibers and demonstrates the attractive potential of PDA coating in fiber-reinforced high-temperature ceramic composites.

## Introduction

Since its first introduction in 2007^1^, polydopamine (PDA) has proven to be a promising multifunctional coating material for a wide range of applications in interface engineering^2–4^. PDA can be prepared by the oxidative self-polymerization of dopamine (DA) under mild alkalinity with the presence of oxygen, producing a uniform and well-bonded coating.

Recently, polydopamine (PDA) has been used to tailor the interface of fiber-reinforced polymer matrix composites^5–13^, significantly enhancing the performance of the composites. Chen et al.^5^ used PDA-modified carbon fibers (C_f_) to reinforce epoxy resin polymer composites. The wetting angle was reduced from 110° to 45° and interfacial bonding was improved, which allowed efficient stress transfer between fibers and matrix, and the tensile strength and Young’s modulus increased by 25% and 34% compared with the unmodified ones. Liu et al.^10^ reported that PDA increased the surface free energy of C_f_ by 27%, making the surface easier to be wetted by polypropylene (PP) resin, resulting in an 88% increase in the interfacial shear strength and an improvement in the mechanical properties of C_f_-reinforced PP composite.

Polydopamine (PDA) also exhibits attractive potential for application in fiber-reinforced high-temperature ceramics composites for the following reasons. Firstly, after carbonization, carbonized polydopamine (C-PDA) possesses a multilayer structure^14,15^. If C-PDA is used as the interfacial phase of fiber-reinforced high-temperature composites, when the composites suffer failure, its laminate structure allows cracks to deflect and branch at the fiber-matrix interfacial region via the peeling and slipping of nanosheets, thereby effectively triggering the toughening mechanisms such as fiber debonding, pulling out, and bridging. Such features facilitate improving damage tolerance of high-temperature ceramics. Secondly, C-PDA can be uniformly and firmly coated on fibers without cross-sticking among adjacent fibers. Without the closed nanoscale pores formed among the fibers which are inevitable in the case of chemical vapor infiltration/deposition (CVI/D)-carbon coating^16,17^, the ceramic matrix could be easily filled among the fibers, and the composite with low porosity could be produced.

However, some key issues limit the application of carbonized polydopamine (C-PDA) in fiber-reinforced high-temperature ceramics composites. On the one hand, the deposition time efficiency of polydopamine (PDA) synthesis is very low. Generally, the substrate is immersed in dopamine (DA)-solution and then PDA coating is formed on the substrate by self-polymerization of DA. However, only tens of nanometers thick PDA coating can be obtained by costing dozens of hours. On the other hand, the thickness of the PDA coating is very small. The thickness of PDA coating could be controlled by the DA-concentration and deposition time. Unfortunately, the maximum thickness of PDA coating obtained in a single reaction step rarely exceeded 100 nm^4,18^. Further increasing DA-concentration or extending the deposition time did not help increase the thickness of PDA coating. What is more, after carbonization, the thickness of C-PDA would decrease to a lower level, which does not meet the requirement of several hundred nanometers thickness for fiber-reinforced high-temperature ceramics composites^16,19^.

For these issues, researchers devoted much effort to developing various methods, such as oxidants catalysis^18,20–24^, ultraviolet (UV) irradiation^25–27^, and cyclic voltammetric (CV) actuation^28–30^. Hong et al.^22^ use sodium periodate (NaIO_4_) as an additive to improve PDA coating kinetics. Based on the optimized dopamine (DA)-concentration and stoichiometric ratio of dopamine to NaIO_4_, the cost time for a 20 nm-thick PDA coating was reduced from 4 h to 1 min. The introduction of oxidizing agents (e.g., NaIO_4_, CuSO_4_, FeCl_3_, H_2_O_2_, O_2_) can accelerate the oxidative self-polymerization reaction of DA, thus improving the deposition time efficiency of PDA^21,23,31^. Taking into account that reactive oxygen species can be generated under UV irradiation, Du et al.^25^ introduced in situ UV irradiation during the coating process to accelerate the polymerization of dopamine. The dehydrogenation of catechol hydroxyl groups was a key step in initiating the polymerization of DA. The cyclic voltammetry (CV) method was developed for the electropolymerization of DA^32–34^. With the assistance of an electric field, catechol hydroxyl groups are more easily deprotonated and the formed negatively charged PDA monomer can be pulled onto the substrate, which can promote the polymerization of PDA. However, in the cyclic voltammetry (CV) method, researchers always control a lower voltage to avoid electrolysis of water. It is believed that the polymerization time efficiency of PDA will be enhanced if the oxygen generated by electrolytic water is used as an oxidizing agent in the polymerization process.

Therefore, in this work, taking into account that hydrolyzed oxygen could be used as a catalyst, an electric field-assisted polymerization (EFAP) route was developed to prepare PDA coating on carbon fibers. The deposition time efficiency of the PDA coating was greatly improved and the thickness limitation was overcome. The thickness of PDA coating could be conveniently designed from nano-scale to micro-scale by adjusting the EFAP parameters, such as dopamine-concentration, current, and deposition time. The effect of EFAP parameters on PDA coating was analyzed. Then PDA coating was pyrolyzed to obtain carbonized polydopamine (C-PDA) coating. The thickness of C-PDA could be tailored by controlling the thickness of PDA. To identify the application of PDA in fiber-reinforced high-temperature ceramics composites, C-PDA was used as an intermediate phase to bond fibers and high-temperature ceramics, with carbon fiber-reinforced ZrB_2_-based composites (C_f_/ZrB_2_-based composite) as an example. The mechanical properties of C_f_/ZrB_2_-based composite were evaluated at room temperature and 1800 °C.

## Materials and methods

### Deposition of PDA coating on carbon fibers by electric field-assisted polymerization

Dopamine hydrochloride (DA, purity: > 98%, supplied by Shanghai Aladdin Bio-Chem Technology Co., Ltd, China) was used as raw material for polydopamine (PDA) coated on the sizing-removed carbon fibers (T700, supplied by Toray Industries, Japan). And tris (hydroxymethyl) aminomethane (Tris, purity: > 98%, supplied by Sinopharm Chemical Reagent Co., Ltd) was used as a buffer. For the electric field-assisted polymerization (EFAP) process, the details were shown below. Carbon fibers were immersed in a DA-Tris aqueous solution (with the concentration ratio of DA and Tris of 2:1), and connected to the positive electrode of the direct current power supply. The graphite plates with a distance of 4 cm were set as symmetric negative electrodes. The EFAP process was carried out at room temperature (RT). The polydopamine (PDA) coating prepared by EFAP was labeled as EFAP-PDA. PDA coating prepared by traditional 24 h self-polymerization (SP) was used as a reference, labeled as SP-PDA. To understand the effect of the EFAP parameters (e.g. dopamine-concentration, current, and deposition time) on PDA coating thickness, Experiments I, II and III were performed, and parameter details were shown in Table 1.

**Table 1:** Experiments for understanding the effect of the EFAP parameters on PDA coating thickness.

Experiments		Conditions								
Experiment I		**Deposition time: 20 min; Deposition current: 300 mA**								
	Dopamine-concentration (mg/ml)	0.125	0.25	0.5	1	1.25	2.5	5	10	20
Experiment II		**Deposition time: 20 min; Dopamine-concentration: 5 mg/ml**								
	Deposition current (mA)	100	150	200	260	300	350	400	450	500
Experiment III		**Dopamine-concentration: 5 mg/ml; Deposition current: 300 mA**								
	Deposition time (min)	5		10		20		40		60

Notation: The current density can be calculated by dividing the current by the area, where the area is 78.72 cm^2^.

### Formation of carbonized PDA coating on carbon fibers

Polydopamine (PDA) coatings obtained from Experiment III (Table 1) with different PDA-deposition times were carbonized to form carbonized polydopamine (C-PDA) coatings on carbon fibers (C_f_). The PDA-coated C_f_ was dried at 80 °C under a vacuum condition and then carbonized in a chamber of a tube furnace at 1200 °C for 1 h in an argon gas atmosphere. The flow rate was 160 ml/min and the heating rate was 4 °C/min. Following this way, the C-PDA coatings with different thicknesses on C_f_ were obtained.

### Fabrication of fiber-reinforced high-temperature ceramics composites

The carbonized polydopamine (C-PDA) coated continuous carbon fibers (C_f_) were used as the reinforcing materials to fabricate unidirectional carbon fiber-reinforced ZrB_2_-based composite (C_f_/ZrB_2_-based composites). The nominal volume fraction of C_f_ was designed at about 40%. ZrB_2_ (purity: > 99.5%, average particle size: 1–3 μm, supplied by Shanghai Aladdin Bio-Chem Technology Co., Ltd, China), ZrSi_2_ (purity: > 99.5%, average particle size: 5 μm, supplied by Shanghai Buhan Chemical Technology Co., Ltd., China) and carbonaceous material (activated charcoal, ≥ 200 mesh, supplied by Shanghai Aladdin Bio-Chem Technology Co., Ltd, China) were used as the raw materials to form the high-temperature ceramics matrix. Where the volume fraction ratio of ZrB_2_ to ZrSi_2_ was 75:25 and the molar ratio of activated charcoal to ZrSi_2_ was 3:1. The green body was fabricated following the procedure described in our previous work^35^. Finally, the green body was hot-pressed at 1600 °C for 20 min with a uniaxial pressure of 40 MPa to obtain C_f_/ZrB_2_-based composites.

### Characterization of liquid samples

After the electric field-assisted polymerization process, the liquid samples were extracted for mass spectrometry (MS) and ultra performance liquid chromatography-mass spectroscopy (UPLC-MS) analysis to understand the synthesis path of polydopamine (PDA). The liquid sample from traditional 24 h self-polymerization was also analyzed and used as a reference. UPLC-MS analysis was carried out on a High-Resolution Orbitrap LC Mass Spectrometer (Exactive GC, Thermo Fisher Scientific, USA). MS analysis was performed on a Q Exactive Plus Mass Spectrometer (Thermo Fisher Scientific, Germany) equipped with an electrospray ionization (ESI) source. For UPLC-MS analysis, the liquid samples were injected into a C18 column eluted at a flow rate of 0.2 ml/min. The mobile phase consisted of water and methanol. The eluate was monitored by an ultraviolet wavelength of 300 nm. The UPLC eluate was collected and pooled for positive ESI‐MS analysis.

### Characterization of coatings

The thicknesses and morphologies of the polydopamine (PDA) and carbonized polydopamine (C-PDA) coatings on carbon fibers (C_f_) were measured and observed by field emission scanning electron microscopes (FE-SEM, NOVA NanoSEM 450, FEI, USA). For each electric field-assisted polymerization (EFAP) condition, at least five filaments were measured to calculate the mean thickness of the coating. The surface chemical compositions of PDA and C-PDA coating on C_f_ were analyzed using X-ray photoelectron spectroscopy (XPS, ESCALAB XI+, Thermo, UK). The structural characterization of C-PDA coating on C_f_ was analyzed using Raman spectroscopy (inVia Qontor, Renishaw PLC, UK) with the excitation wavelength of 532 nm.

### Characterization of composite

The density of the composite was measured by Archimedes’ method. The morphologies of polished and fractured surfaces of the composite were analyzed by field emission scanning electron microscope (FE-SEM, NOVA NanoSEM 450, FEI, USA). Single fiber push-out test was performed using a Nanomechanical Testing System (TI 950, Hysitron, USA) to understand the failure mode of the fiber-matrix interface.

The flexural strength of composites was evaluated by a 3-pt flexural test with the test bars of 25 mm × 2.5 mm × 2 mm (length by width by thickness). According to ASTM C1211-18, the cross speed at room temperature (RT) and 1800 °C was set as 0.5 mm/min and 3 mm/min, respectively. According to the ASTM C1421-18, the fracture toughness was evaluated by single edge-notched beams (SENB) test with a crosshead speed of 0.05 mm/min. The SENB test bars were 22 × 2 × 4 mm^3^ (length by width by thickness) with a notch of 2 mm depth. Each test was repeated on three bars. The high-temperature test was carried out under a vacuum condition. The heating profile was 15 °C/min to 1800 °C and then follows by a 5-min isothermal hold to allow equilibration of the bars and fixture at the test temperature. After the test, the bars were cooled with the furnace.

To obtain a more accurate work of fracture (*WOF*), an extensometer (equipped with S-series displacement transducers of Solartron Metrology, with 0.001 mm resolution) was used to measure the displacement. The *WOF* was calculated using the following Eq. (1):

1 $$WOF = \frac{W}{2A}$$

where *W* was the area under the load–displacement curves and *A* was the area of the cross-section of the specimens.

## Results and discussion

### Deposition of PDA coating on carbon fibers by EFAP

**Figure 1:**
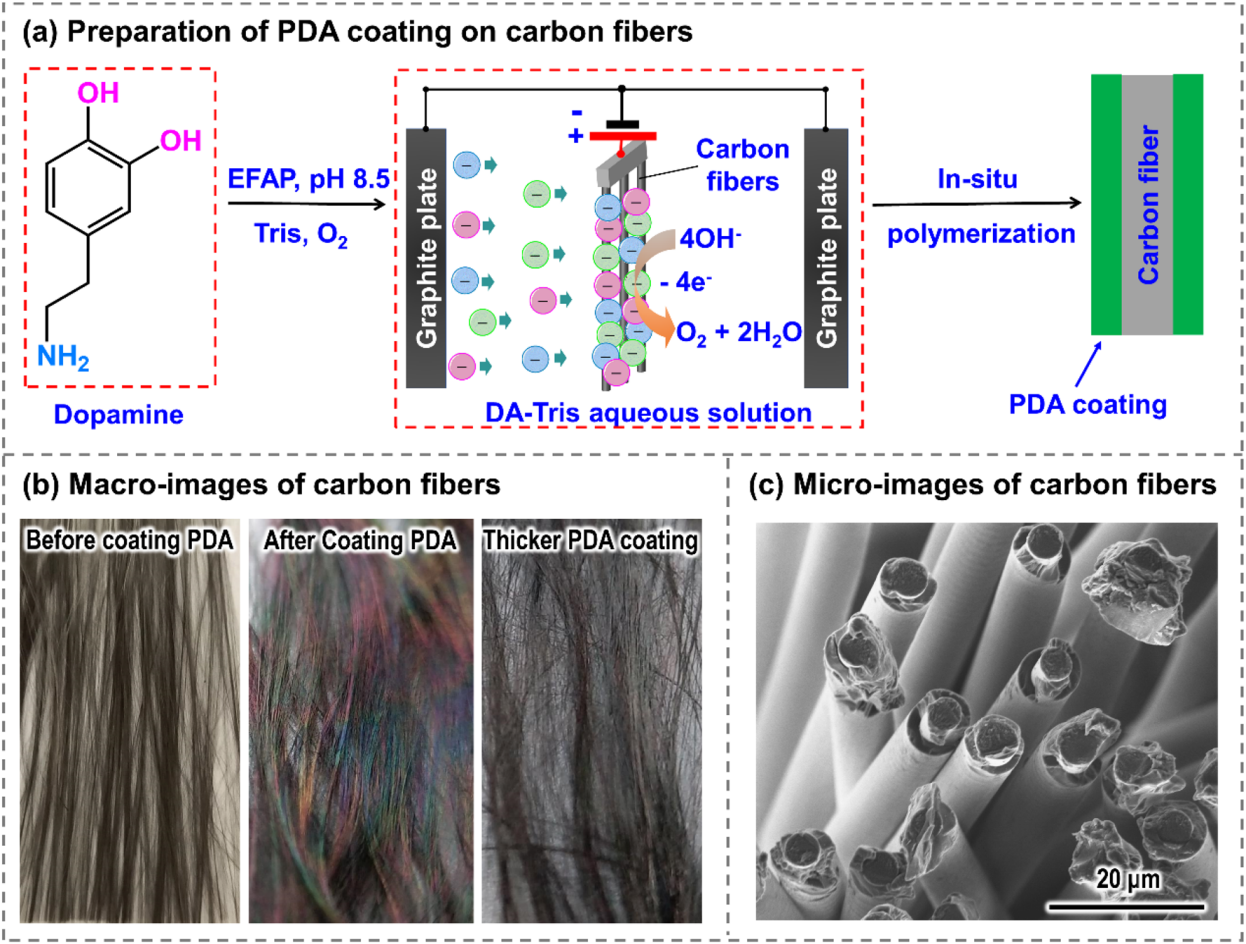
(**a**) Scheme for the preparation of PDA coating on carbon fibers deposited by electric field-assisted polymerization. (**b**) Macro-images of carbon fibers before and after coating PDA. (**c**) SEM images of PDA-coated fibers show no visible cross-sticking in an intra-bundle.

Figure 1a illustrates the schematic diagram for the preparation of polydopamine (PDA) coating on carbon fibers (C_f_) via electric field-assisted polymerization (EFAP). Under the action of the electric field, the OH^-^ loses electrons on the anode C_f_ and produces oxygen, which could promote the oxidation reaction of dopamine. Negatively charged PDA monomers can be obtained by deprotonating dopamine and its derivatives. These negatively charged PDA monomers aggregates around C_f_ and lose electrons, forming a PDA coating via in-situ polymerization. Before the EFAP process, the surface of C_f_ appears light gray as displayed on the left of Fig. 1b. After the EFAP process, the surface color of C_f_ changes to iridescent as shown in the middle of Fig. 1b, indicating that PDA coating has been successfully coated on C_f_. As the thickness of PDA coating increases, the surface color of C_f_ changes from iridescent to black, as shown on the right of Fig. 1b. By using EFAP, a uniform and dense PDA coating was coated on C_f_ with no visible cross-sticking in an intra-bundle (Fig. 1c).

### The effect of EFAP parameters on PDA coating

Generally, the thickness of polydopamine (PDA) coating prepared by the traditional self-polymerization (SP) rarely exceeds 100 nm^4,18^, accompanied by a cost of dozens of hours. Fascinating, we easily realized a uniform and dense PDA coating with a micro-thickness in a short time via electric field-assisted polymerization (EFAP). The thickness of PDA coating could be conveniently designed from nano-scale to micro-scale by adjusting the EFAP parameters, such as dopamine-concentration, current, and deposition time. The effect of EFAP parameters on PDA coating was analyzed.

**Figure 2:**
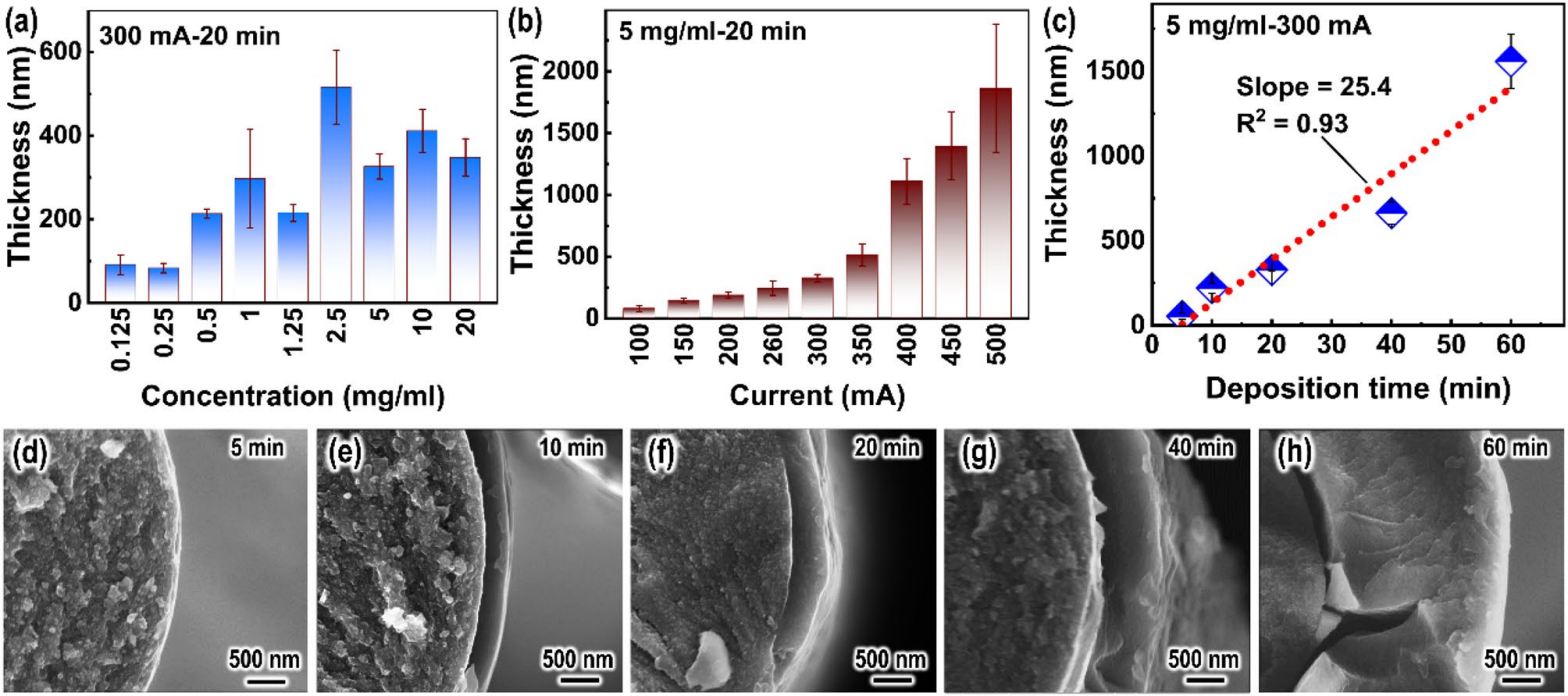
PDA coating thickness as a function of (**a**) DA-concentration (with a current of 300 mA and a deposition time of 20 min), (**b**) current (with a DA-concentration of 5 mg/ml and a deposition time of 20 min), and (**c**) deposition time (with a DA-concentration of 5 mg/ml and a current of 300 mA). (**d**–**h**) SEM images of PDA coatings deposited at different deposition times (with a DA-concentration of 5 mg/ml and a current of 300 mA). The different deposition times have been marked.

Figure 2a shows the thickness of polydopamine (PDA) coating on carbon fibers as a function of dopamine (DA)-concentration. Here, PDA coatings were fabricated from Experiment I (Table 1). The DA-concentration was within 0.125–20 mg/ml, with a current of 300 mA and a deposition time of 20 min. The mean thickness of the PDA coating increases nonlinearly from 91 to 516 nm when the DA-concentration increases from 0.125 to 2.5 mg/ml. Subsequently, the thickness of the PDA coating decreases to 326 nm at 5 mg/ml, and no obvious change is observed even though the DA-concentration increases again. The PDA coating thickness fluctuates around ~ 350 nm at the range of 5–20 mg/ml. The reason for the non-linearity of PDA coating thickness with DA-concentration would be further analyzed in the next section.

Figure 2b shows the thickness of polydopamine (PDA) coating on carbon fibers as a function of current. Here, PDA coatings were fabricated from Experiment II (Table 1). The current was within 100–500 mA, with a dopamine-concentration of 5 mg/ml and a deposition time of 20 min. It can be seen that the mean thickness of the PDA coating increases from 78 nm up to 1863 nm when the current changes from 100 to 500 mA. In addition, when the current exceeds 300 mA, the thickness of the PDA coating increases faster. This may be attributed to the higher voltage promoting the electrolysis of water, which produced more oxygen and thus promoted the polymerization of dopamine. The reasons for the improvement in deposition rate would be further analyzed in the next section.

Figure 2c shows the thickness of polydopamine (PDA) coating on carbon fibers (C_f_) as a function of deposition time. Here, PDA coatings were fabricated from Experiment III (Table 1). The deposition time was within 5–60 min, with a dopamine-concentration of 5 mg/ml and a current of 20 min. As can be seen, the thickness of PDA coating almost linearly increases with increasing deposition time. The fitting line denoted by dashed one presented a slope of 25.4, indicating a high deposition rate. Figure 2d–h shows the scanning electron microscope (SEM) images of PDA coatings deposited at different times. With a deposition time of 5 min (Fig. 2d), a 54 nm PDA coating is wrapped around C_f_, indicating that PDA is successfully deposited on it. When the deposition time is increased to 60 min, the thickness of PDA coating is up to 1558 nm as shown in Fig. 2h. Again, the PDA coatings are uniform and dense, even for a very thick coating, which is unlike the work reported in the literature that the coating is a loose accumulation of particles^13,36^.

**Figure 3:**
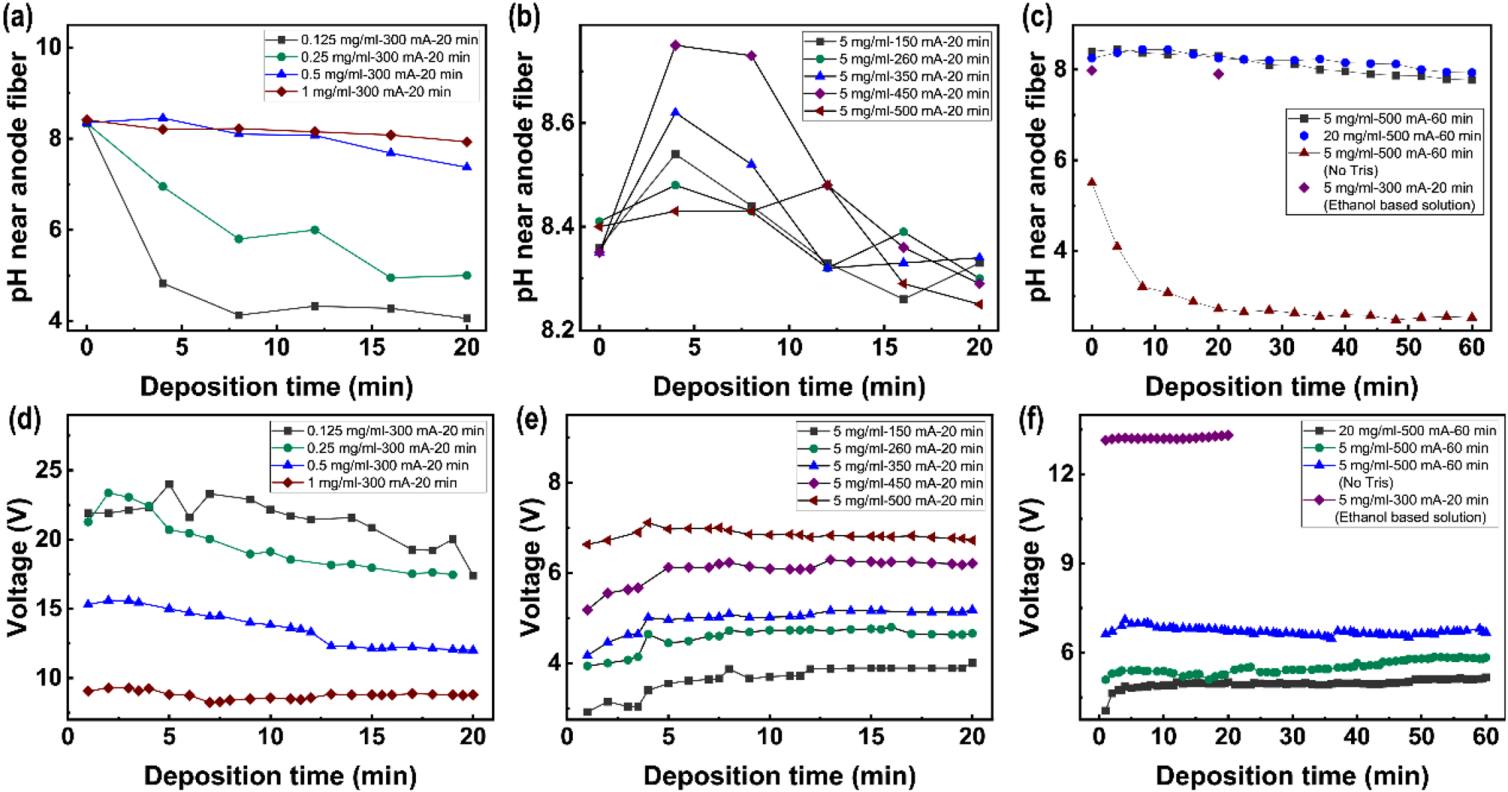
The pH near anode fiber and voltage during the electric field assisted polymerization (EFAP) process deposited at: (**a**,**d**) different DA-concentration, (**b**,**c**) different current, (**c**,**f**) high current, high DA-concentration with and without Tris in the water-based solution. Deposition was also performed in an ethanol-based solution. EFAP parameters have been marked. For example, EFAP parameter for a DA concentration of 5 mg/ml, a current of 500 mA, and a deposition time of 60 min are labeled as 5 mg/ml-500 mA-60 min.

The favorable condition for the polymerization of dopamine (DA) into polydopamine (PDA) is an oxygen-rich alkaline environment^1,18,22^. Therefore, the pH of the solution and oxygen from water electrolysis play important roles in the deposition of PDA coating. To reveal the intrinsic mechanism of PDA thickness as a function of electric field-assisted polymerization (EFAP) parameters, the pH and voltage were monitored. The results were summarized in Fig. 3. Figure 3a–c and Fig. 3d–f show the pH and voltage, respectively.

The alkaline environment is able to absorb the protons from dopamine (DA) and its derivatives, thus accelerating the polymerization reaction of PDA^22,32,34^. Here Tris was used as a buffer to adjust the pH, and the Tris-concentration was set to half of DA. The pH near the anode fiber tends to be slightly higher than that of the cathode graphite plate due to electrostatic attraction (Fig. 1S). At lower Tris-concentration of 0.0625 mg/ml (with DA-concentration of 0.125 mg/ml), the pH near anode fiber decreased from 8.33 to 4.06 with increasing deposition time to 20 min (Fig. 3a). The variation of the pH with DA-concentration is one reason for the non-linearity of PDA coating thickness with DA-concentration (Fig. 2a). Deposition without Tris in the solution was also performed and pH near anode fiber decreased to 2.53 as the deposition time increased to 60 min (Fig. 3c). These results suggested that the electric field promoted the dehydrogenation of DA and its derivatives, which was consistent with the results in literatures^28,30,32,34^. Tris can provide an alkaline environment to absorb protons from DA and its derivatives, which is important for the deposition of PDA. When Tris-concentration exceeds 0.5 mg/ml, the pH of the solution is maintained in the range of 7.82–8.75 during the deposition process (Fig. 3a–c). The PDA coating with a thickness of 2148 nm (Fig. 2Sa) was obtained by depositing at a DA-concentration of 5 mg/ml (with Tris-concentration of 2.5 mg/ml), a current of 500 mA and a deposition time of 60 min. On the contrary, for deposition without Tris in the solution, the thickness of PDA coating was only 219 nm (Fig. 2Sb). DA and its derivatives tend to be negatively charged due to dehydrogenation, which aggregate to the anode fiber under the action of the electric field and polymerize in situ to form a PDA coating. To confirm this, the deposition of PDA on carbon fibers (C_f_) was carried out at the cathode in a water-based solution (with a DA-concentration of 5 mg/ml, a current of 400 mA and a deposition time of 20 min). As shown in Fig. 2Sc, almost no PDA coating was deposited on C_f_, in contrast to the anodically deposited 1110 nm thick PDA coating (Fig. 2b).

High voltage can provide more hydrolyzed oxygen that can act as a catalyst, which is beneficial to the deposition of polydopamine (PDA). The effect of hydrolysis oxygen is another reason for the non-linearity of PDA coating thickness with dopamine (DA)-concentration. At lower solution concentrations, the voltage between electrodes is higher under constant current conditions due to the low concentration of ions in the solution and the poor conductivity of the solution. For example, although the pH at 0.125 mg/ml was lower than that at 0.25 mg/ml (Fig. 3a), the voltage at 0.125 mg/ml was higher than that at 0.25 mg/ml (Fig. 3d), and thus the thickness of the PDA coating at 0.125 mg/ml was not smaller than that at 0.25 mg/ml (Fig. 2a). Also, at higher solution concentrations, with the pH around ~ 8.4, the voltage decreased and the thickness of PDA coating did not continue to increase (Fig. 2a). In order to obtain thicker coatings, the current can be appropriately increased to obtain higher voltages. For deposition at different currents (with a DA-concentration of 5 mg/ml and a deposition time of 20 min), the voltage increases with increasing current (Fig. 3e). A significant increase in the gas produced was observed at 300 mA. This is consistent with the increase in thickness of PDA coating with increasing current in Fig. 2b. DA-concentration above 2.5 mg/ml, the voltage and pH remains stable during the deposition process (Fig. 3b,c,e,f). Thus the thickness of PDA coating linearly increases with deposition time (Fig. 2c). To confirm the contribution of hydrolyzed oxygen, the deposition of polydopamine (PDA) on carbon fibers (C_f_) was performed in an ethanol-based solution (with a dopamine (DA)-concentration of 5 mg/ml, a current of 300 mA and a deposition time of 20 min). Almost no oxygen was produced during the deposition process. The resulting thickness of PDA coating was about 30 nm (Fig. 2Sd), which was much lower than the 326 nm deposited in a water-based solution.

In short, it is the first time achieving a micro-scale polydopamine (PDA) coating. The deposition rate of PDA coating is up to 5589 nm/h at 500 mA (with a dopamine-concentration of 5 mg/ml and a deposition time of 20 min), which is much higher than that of other methods. To confirm the improvement of the electric field-assisted polymerization (EFAP) method in this work, Table 2 shows the deposition time efficiency of PDA coating prepared with different methods. As can be seen, the deposition rate of the EFAP method is 3 orders of magnitude larger than self-polymerization^1^ and ultraviolet-triggered method^25,26^, also 2 orders of magnitude larger than oxidants-induced method^20,21^. Noting that the thickness of PDA only reaches 2755 nm (Fig. 2Se) after depositing at 500 mA for 2 h (with a dopamine-concentration of 5 mg/ml) due to the consumption of DA during the deposition process (Fig. 3S). Nonetheless, the thickness of PDA in this work is much higher than that in other methods, implying that the thickness limit is overcome. These results demonstrate that EFAP is capable of ultrafast deposition of PDA coating.

**Table 2:** Comparison for the deposition time efficiency of PDA coating prepared with different methods.

Methods	Deposition time (min)	Thickness (nm)	Deposition rate (nm/h)	Thickness limitation (nm)	References
Self-polymerization	1440	50	2	Rarely exceeded 100 nm in a single deposition step	^1^
(NH_4_)_2_S_2_O_8_-induced	120	70	35		^20^
CuSO_4_/H_2_O_2_-induced	40	30	45		^21^
FeCl_3_/H_2_O_2_-induced	40	75	113		^23^
Pure O_2_-induced	30	4	8		^31^
UV-triggered	120	4	2		^25^
UV-triggered	180	11	4		^26^
EFAP	20	1863	5589	2755	This work

### Synthesis path of PDA

**Figure 4:**
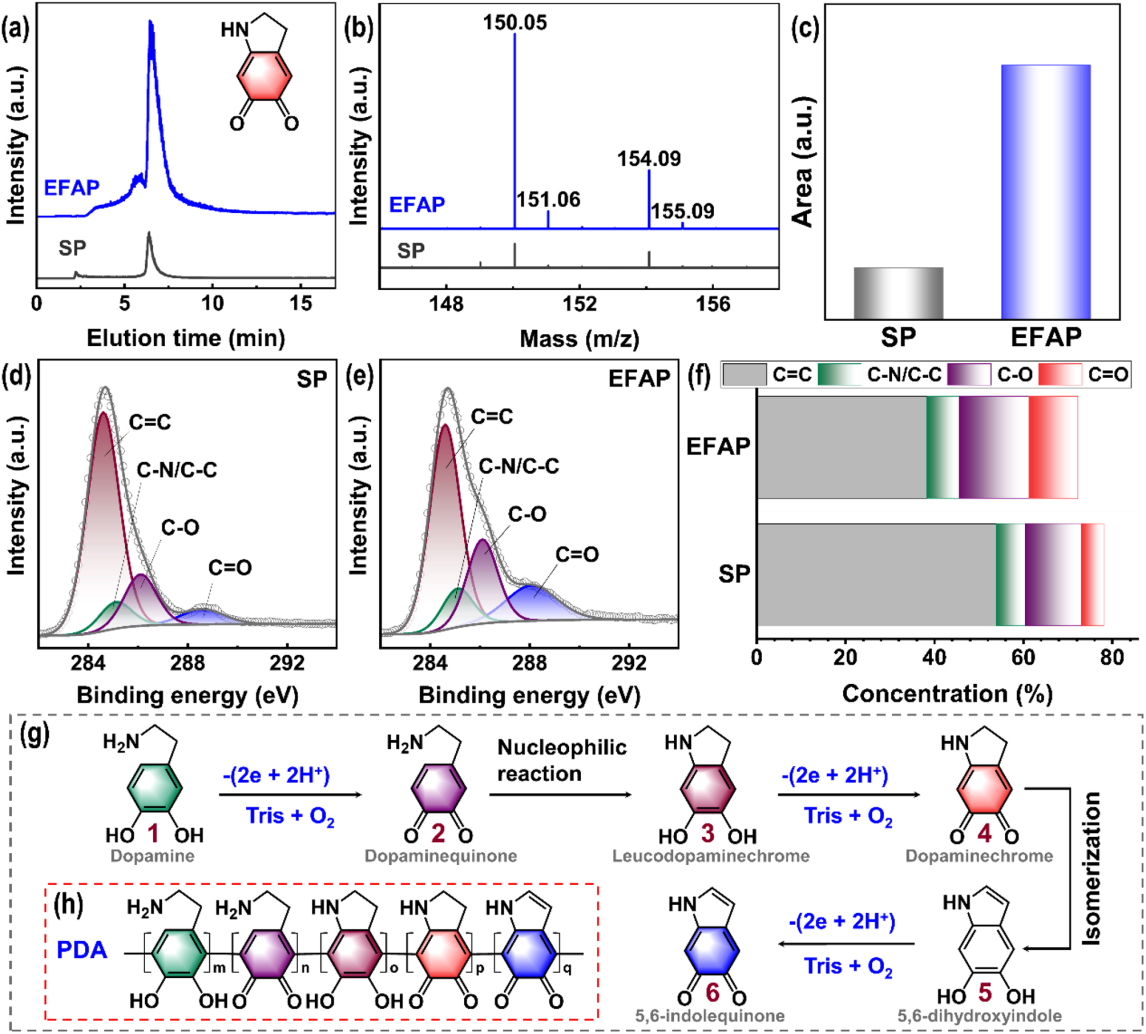
UPLC-MS analysis for liquid samples from self-polymerization (SP) and electric field-assisted polymerization (EFAP) samples: (**a**) chromatograms of dopaminechrome, (**b**) ESI-MS analysis, and (**c**) the area from chromatographic peaks. The high-resolution C1s XPS spectra of (**d**) SP-PDA, (**e**) EFAP-PDA, and (**f**) XPS atomic concentration of functional groups. Scheme for (**g**) the conversion path of DA to its derivatives and (**h**) the structure of PDA.

To further understand the enhanced deposition rate of polydopamine (PDA) coatings via electric field-assisted polymerization (EFAP), mass spectrometry (MS) and ultra performance liquid chromatography-mass spectroscopy (UPLC-MS) analyses were performed on the solutions from traditional 24 h self-polymerization (SP) and electric field-assisted polymerization (EFAP, with a DA-concentration of 5 mg/ml, a current of 200 mA or 400 mA, and a deposition time of 20 min). The dopaminechrome was identified by a chromatographic peak with an elution time of 6.44 min (Fig. 4a) and a strong [M + H]^+^ ion signal at m/z 150.05 (Fig. 4b). The concentration of dopaminechrome in the solution largely determines the deposition time efficiency of PDA coating on the material^37–39^. The limited thickness of PDA prepared by SP is due to the low conversion efficiency of dopamine (DA) to dopaminechrome and the sequestration of dopaminechrome by PDA particles^38^. In this work, EFAP greatly promotes the conversion of dopamine to dopaminechrome, as the area of the chromatographic peak of EFAP (liquid sample from EFAP with a DA-concentration of 5 mg/ml, a current of 400 mA and a deposition time of 20 min) is 5.3 times that of SP (liquid sample from SP with a DA-concentration of 5 mg/ml and a deposition time of 24 h) as shown in Fig. 4c, which is favorable for the efficient synthesis of PDA. And the chromatographic peak area of EFAP at 400 mA is 8% higher than that at 200 mA (liquid sample from EFAP with a DA-concentration of 5 mg/ml and a deposition time of 20 min), indicating that the conversion efficiency increases with increasing current, which confirms the results in Fig. 2b. Figure 4d–e shows the high-resolution C1s X-ray photoelectron spectroscopy (XPS) spectra of PDA coatings prepared by EFAP (samples from EFAP with a DA-concentration of 5 mg/ml, a current of 400 mA and a deposition time of 20 min) and SP. It can be seen that the main composition is the C=C bond, corresponding to the aromatic ring. EFAP-PDA possesses more oxygen content (Fig. 4S). Also, EFAP-PDA possesses more C–O and C=O bonds compared to SP-PDA, as shown in Fig. 4f, implying more DA-oxidized derivatives.

**Figure 5:**
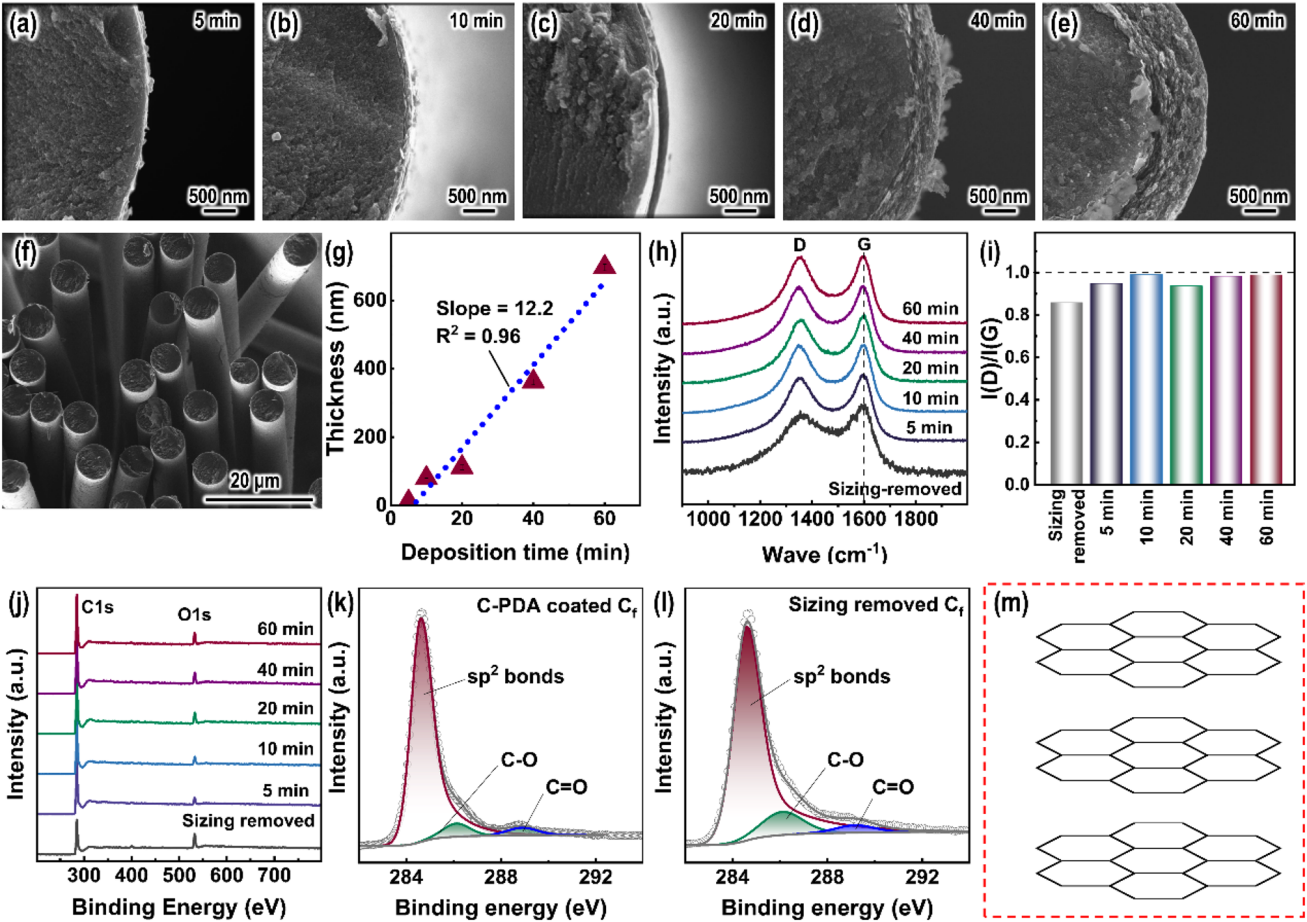
SEM images for (**a**–**e**) C-PDA coatings with different PDA-deposition times, (**f**) no visible cross-sticking in an intra-bundle. (**g**) The thickness of C-PDA coating as a function of PDA-deposition time. (**h**) Raman spectra of C-PDA coatings with different PDA-deposition times and (**i**) the intensity ratio of D- and G-band. (**j**) XPS survey spectra of C-PDA coatings with different PDA-deposition times, and the high-resolution XPS spectra: (**k**) C1s of C-PDA coatings with the PDA-deposition times of 60 min, (**l**) C1s of sizing removed carbon fibers. (**m**) Scheme for the structure of C-PDA coating. The different PDA-deposition times from the EFAP process have been marked.

**Figure 6:**
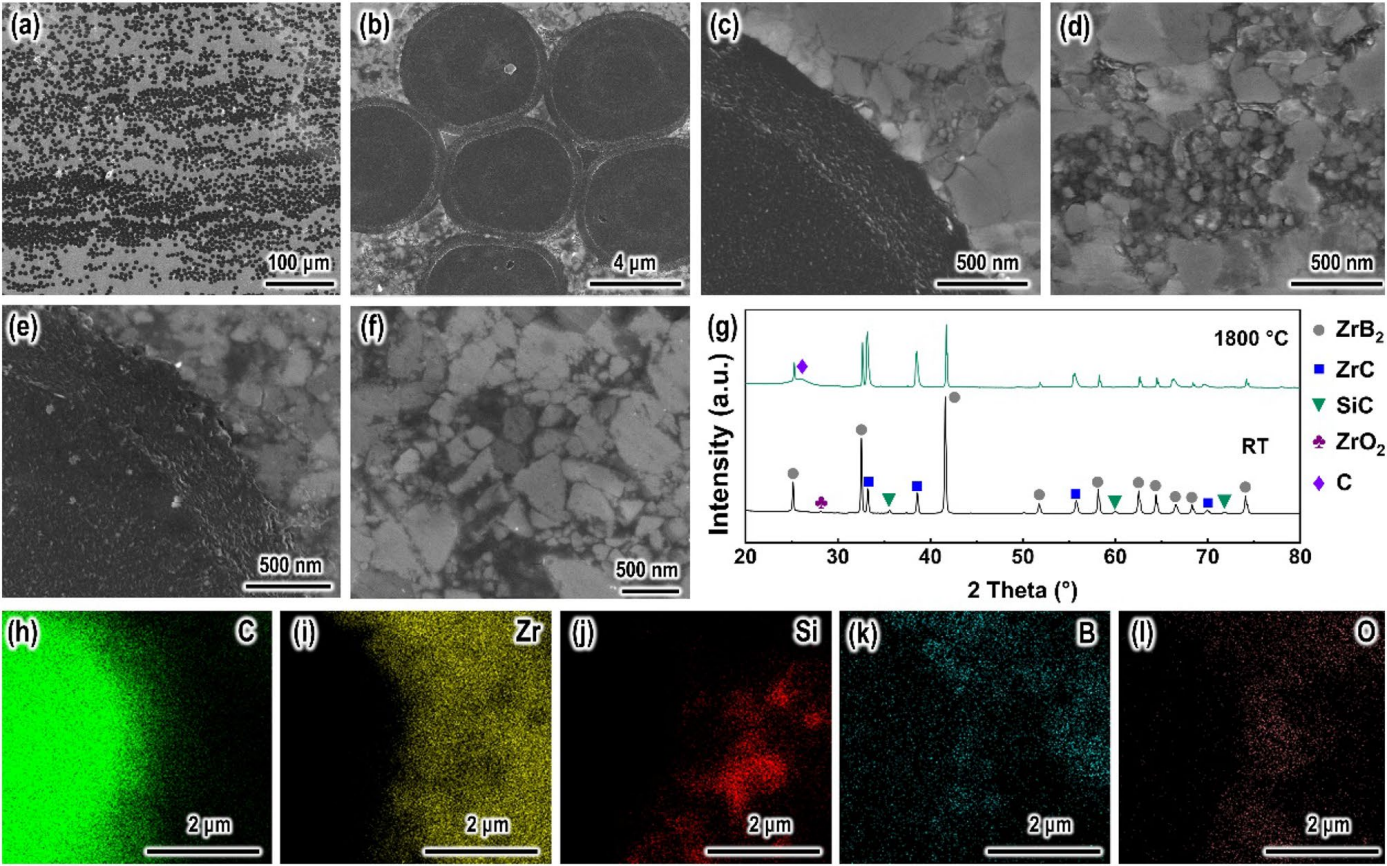
SEM images of C-PDA coated carbon fiber reinforced high-temperature composite: (**a**,**b**) polished cross-section showing fibers well distrusted in the matrix, high magnification view of (**c**) fiber-matrix interface region and (**d**) matrix, and high magnification view of (**e**) fiber-matrix interface region and (**f**) matrix after heat treatment at 1800 °C for 50 min. (**g**) X-ray diffraction patterns of the composite before and after heat treatment at 1800 °C for 50 min. (**h**–**l**) EDS mapping for fiber-matrix interface region after heat treatment at 1800 °C for 50 min. Elements have been marked.

**Figure 7:**
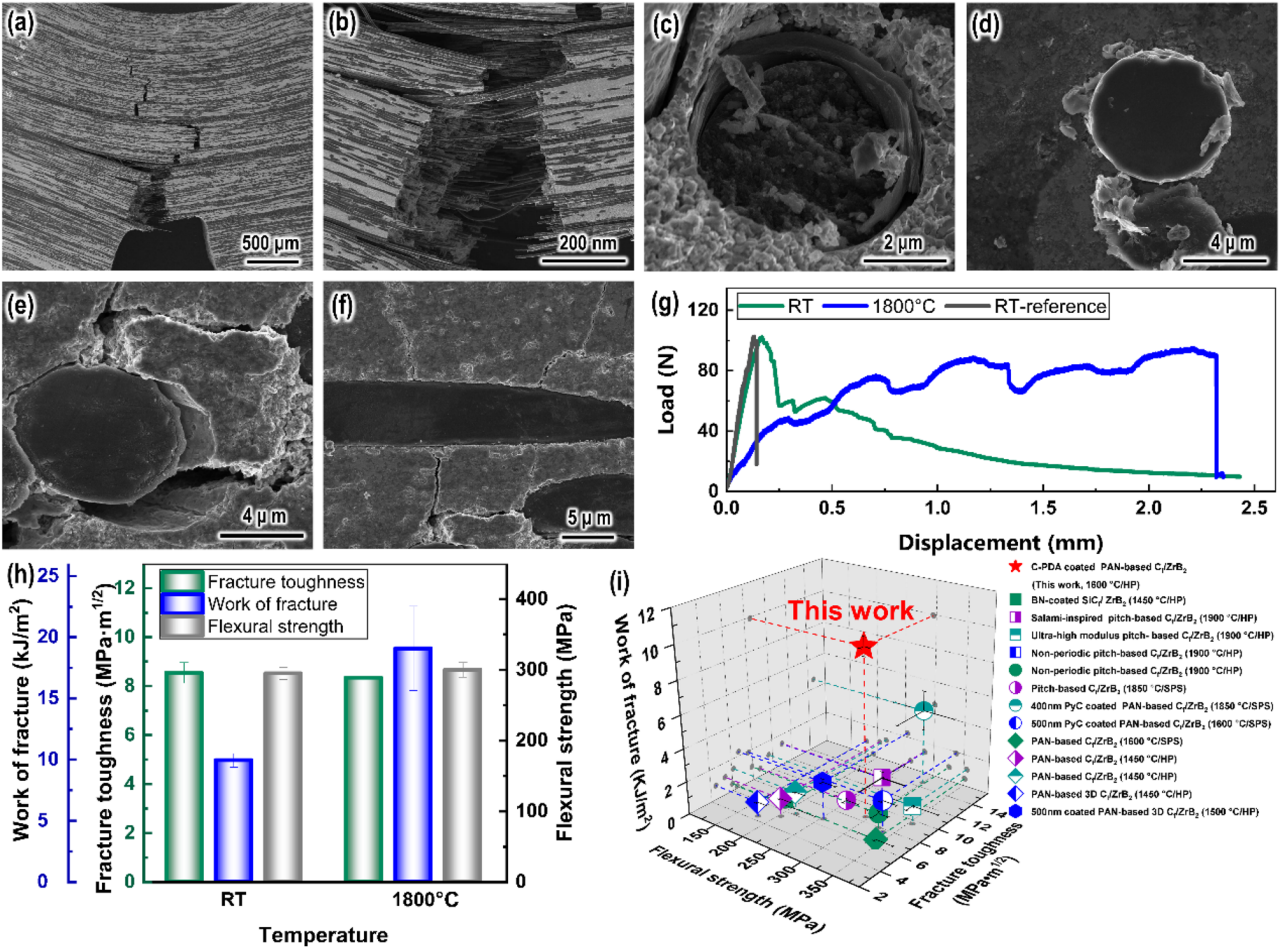
Morphologies of the composite after SENB test at 1800 °C: (**a**) tortuous crack propagation path, (**b**) fibers pull-out. (**c**) C-PDA peels off like an onion. (**d**) Pushed-out single fiber by the nano-indentation technique showing the peeling of C-PDA. (**e**,**f**) Cracks propagation around the fiber-matrix region. (**g**) Load–displacement curves after SENB test, non-C-PDA coated one^35^ as a reference. (**h**) Properties of the composite at RT and 1800 °C. (**i**) The data for flexural strength, fracture toughness and work of fracture^16,19,43–50^. Figure (**g**–**i**) was plotted by OriginPro (2022b (64-bit), 9.9.5.167 (Learning Edition), https://www.originlab.com/).

The electric field-assisted polymerization (EFAP) process can accelerate the conversion of dopamine (DA) to its oxidized derivatives, thereby accelerating the synthesis of polydopamine (PDA). Figure 4g shows a scheme for the conversion path of DA to its derivatives. The oxidation process consists of 3 stages. First, DA is oxidized to dopaminequinone. Dopaminequinone is susceptible to nucleophilic reactions and forms intramolecular rings via 1,4-Michael addition, resulting in the more easily oxidized leucodopaminechrome. Second, leucodopaminechrome is oxidized to dopaminechrome. Dopaminechrome can further isomerize to 5,6-dihydroxyindole. Third, 5,6-dihydroxyindole is oxidized to 5,6-indolequinone. The EFAP method in this work played a significant role in promoting these three stages. The deposition of PDA on carbon fibers was carried out at the anode, where OH^-^ lost electrons to produce oxygen to create an oxygen-rich environment. Also, the H^+^ lost from DA and its derivatives would not only be consumed by Tris, but also swam to the cathode and escaped as H_2_ gas. Therefore, the oxidation reaction tended to move to the right. Based on mass spectrometry (MS) analysis, some oligomers were detected, as shown in Figs. 5S–9S. The deprotonated DA and its oxidized derivatives aggregated toward carbon fibers (C_f_) anode driven by an electric field and underwent in-situ self-polymerization to form oligomers. Then these PDA oligomers form a dense PDA coating on C_f_ (Fig. 2d–h) through π–π interactions and further polymerization^40^. Compounds **1**, **2**, **3**, **4**, and **6** were the partial building blocks of PDA. Figure 4h shows the scheme for the structure of PDA.

### Carbonized PDA coating and its structural characterization

Polydopamine (PDA) coatings obtained from Experiment III (Table 1) with different deposition times were pyrolyzed to form carbonized polydopamine (C-PDA) coatings on carbon fibers (C_f_). From scanning electron microscope (SEM) observation in Fig. 5a–e, C-PDA coating was still tightly wrapped around C_f_, and C-PDA coating exhibits a layered structure. Also, there is no visible cross-sticking of neighboring fibers in a bundle as shown in Fig. 5f and Fig. 10Sa. The flat surface along the length of C_f_ indicates that C-PDA coating on C_f_ is continuous and uniform (Fig. 10Sa,b). The multilayer structure of C-PDA coating can be further observed in Fig. 10Sc–e, with the thickness of the layer of ~ 5.5 nm. The thickness of C-PDA coatings with different PDA-deposition times varies from 11 to 698 nm, which is quite dependent on the thickness of PDA coatings.

Figure 5g shows the thickness of carbonized polydopamine (C-PDA) coating as a function of PDA-deposition time. The thickness of C-PDA coating increases with increasing PDA-deposition time. The slope of the fitting line based on the measured C-PDA thickness is about 12.2. Compared with Fig. 2c, the slope in Fig. 2c is almost twice that of Fig. 5g, implying that the thickness retention of the PDA coating after carbonization is up to 50%. Indeed, comparing the scanning electron microscope (SEM) images in Figs. 2d–h and 5a–e, the thickness of C-PDA coating is about half of that of polydopamine (PDA) coating. The shrinkage of PDA coating after carbonization was attributed to the release of N, O, and H elements. Also, part of the C elements was released during pyrolysis as small molecules and CO. The higher residual carbon rate was due to the high density and high carbon content of PDA coating.

To understand the structural characterization of carbonized polydopamine (C-PDA) coating, Raman spectroscopy and X-ray photoelectron spectroscopy (XPS) analysis were performed. Figure 5h shows the Raman spectrum of C-PDA coating. Two dominating peaks appear at 1350 and 1600 cm^−1^, corresponding to the D- and G-band, respectively. The intensity ratio of D- and G-band of C-PDA coating are below 1.00, closing to 0.86 for sizing removed carbon fibers (Fig. 5i). These results suggested that the C-PDA coating contained some rings and possessed an ordered carbon structure. Figure 5j shows the XPS survey spectrums for the C-PDA coatings. The C1s peak around 285 eV and O1s peak around 531 eV were revealed. The surface chemical structure of C-PDA coating was further investigated via high-resolution XPS spectra. From the C 1s of C-PDA coating (Fig. 5k), the C 1s could be decomposed into sp^2^ bonds, C–O bonds and C=O bonds, which is similar to sizing removed carbon fibers (Fig. 5l). The large number of aromatic rings of PDA and the π–π interaction stacking of PDA oligomers tend to produce a graphite-like layered structure^14,15,40^, which confirms the main composition of sp^2^ bonds. Based on the above analysis, Fig. 5m presents a scheme for the structure of C-PDA coating, showing a layered structure.

In short, carbonized polydopamine (C-PDA) coatings can be efficiently prepared following the processing route in this work. The main features of the current processing route are: the homogeneous C-PDA coatings are formed on carbon fibers (C_f_) not only at the surface of the bundles but also at the center of the bundles; there is no visible cross-sticking of neighboring fibers in a bundle; the thickness of C-PDA coating could be easily controlled by adjusting PDA-deposition time. What's more, the C-PDA coating exhibits a layered structure. If C-PDA coating is used as an interphase of fiber-reinforced high-temperature ceramics composites to bond rigid fiber and brittle ceramics, the toughening mechanism of the fibers would be easily triggered, and the damage tolerance of the composites would be improved.

### Properties of carbon fiber-reinforced high-temperature ceramics composites

To identify that the application of polydopamine (PDA) can be extended to fiber-reinforced high-temperature ceramics composites, carbonized polydopamine (C-PDA) coating with a mean thickness of ~ 500 nm was used as the interphase of carbon fiber-reinforced ZrB_2_-based composites (C_f_/ZrB_2_-based composite) to bond fibers and ZrB_2_-based ceramics.

As carbonized polydopamine (C-PDA) coating was uniformly coated on carbon fibers (C_f_) without visible cross-sticking among neighboring fibers, the ceramic slurry well infiltrated into the fiber bundles. The C_f_/ZrB_2_-based composite possesses a homogeneous fiber-matrix distribution with almost no micropores observed, as shown in Fig. 6a,b. Based on Archimedes’ method, the porosity of the composite was measured at 6%, which was much lower than ~ 18%^16,17^. The lower porosity in this work was attributed to the good infiltration of the ceramic slurry in fiber bundles and the promotion of densification by ZrSi_2_. Specifically, ZrSi_2_ has a low yield strength above 1200 °C^41^ and is able to deform plastically under sintering pressure to promote the rearrangement of ceramic particles and reduce porosity^42^. As a result, a near fully dense composite can be obtained under mild conditions.

C-PDA coating can bond fiber and matrix well. As shown in Fig. 6b,c, C-PDA coating is tightly bonded to fiber and matrix and is not peeled off like chemical vapor infiltration/deposition (CVI/D)-carbon coating^16,17^ under uniaxial loading during high-temperature sintering. In addition, due to the protection of activated charcoal, C-PDA coating did not suffer from severe corrosion of ZrSi_2_. As shown in Fig. 6g, after sintering, ZrSi_2_ was not detected by X-ray diffraction and was replaced by newly formed ZrC and SiC. Also, the enrichment of Si at the C-PDA coating-matrix interface was also not observed by energy dispersive X-ray spectroscopy (EDS, Fig. 11S). This implied that ZrSi_2_ tended to react with activated charcoal in the matrix rather than C-PDA to produce nanoscale ZrC and SiC clusters (grain size ~ 31 nm, Fig. 6d). Further, the sintered sample was heat treated at 1800 °C for 50 min. The crystallinity of ZrC and SiC increased (Fig. 6g) and the grains grew to ~ 131 nm (Fig. 6f). EDS analysis shows no significant corrosion of C-PDA coating (Fig. 6h–l). C-PDA coating maintains a tight bond with the fiber and matrix after heat treatment at 1800 °C for 50 min (Fig. 6e). These results facilitate a good mechanical coupling between the fiber and matrix.

Furthermore, carbonized polydopamine (C-PDA) coating with a layered structure is prone to deflection cracks, thus improving the fracture resistance of the composite. As shown in Fig. 7a,b and Fig. 12Sa,b,e,f, the composite exhibits significant non-brittle fracture characteristics. From Fig. 7a and Fig. 12Sa, cracks mostly propagate forward along a zigzag path and even expand into a network. Extensive crack branch and crack deflection can be observed. From Fig. 7b and Fig. 12Sb, a large number of fibers are pulled out. The interlayer bonding of C-PDA is weak. The layers within C-PDA can peel off when the cracks extend to the fiber-matrix interface region. As shown in Fig. 7c, C-PDA is observed to peel off like an onion on the fractured fiber. And it can be observed in Fig. 12Sc,d that C-PDA causes the fibers to slide by peeling off the weak layer. To further understand the failure characteristics within the fiber-matrix interface domain, single fiber was pushed out using nano-indentation technology. As shown in Fig. 7d, a large number of lamellar fragments exfoliated from C-PDA can be observed. The interlayer peeling of C-PDA can passivate the crack tip and redistribute the stress at the interface, thus causing crack branching and crack deflection (Fig. 7e,f). These results help to absorb the fracture energy and improve the fracture properties of the composite.

Carbonized polydopamine (C-PDA) coating can significantly improve the damage tolerance of C_f_/ZrB_2_-based composite. Figure 7g shows the typical load–displacement curve of the composite acquired from the SENB test. Significant pop-in and pseudo-ductility behavior can be observed in C-PDA coated C_f_/ZrB_2_-based composite, exhibiting non-brittle failure mode with an extension of deflection up to ~ 2.3 mm. In contrast, for the non-C-PDA coated fiber-reinforced high-temperature composite^35^, the load rapidly drops to a very low level after reaching the maximum, implying lower damage tolerance. Figure 7h summarized the flexural strength, fracture toughness and work of fracture of the C-PDA coated carbon fiber reinforced high-temperature composite. The flexural strength of 300 ± 10 MPa and fracture toughness of 8.34 ± 0.05 MPa·m^1/2^ were achieved at 1800 °C and remained nearly constant compared to that at room temperature, demonstrating good load-bearing capacity and thermal stability. Also, C-PDA coated C_f_/ZrB_2_-based composite exhibited extraordinary work of fracture of 9936 ± 548 J/m^2^ at RT and 19,082 ± 3458 J/m^2^ at 1800 °C, exhibiting good damage tolerance.

To clarify the position of the properties of the composites, the data were compared with those reported in the literature. Figure 7i shows the data for room temperature (RT) properties of fiber/ZrB_2_-based composite^16,19,43–50^. As can be seen, the carbonized polydopamine (C-PDA) coated C_f_/ZrB_2_-based composite possesses competitive strength and toughness. For example, the flexural strength of pich-based C_f_/ZrB_2_-based composite was 235 MPa at RT and 240 MPa at 1800 °C^51^. Carbon coating was shown to be able to modulate the fiber-matrix bonding well to balance the trade-off between strength and toughness. The flexural strength and fracture toughness of C_f_/ZrB_2_-based composite can reach 631 MPa and 15.7 MPa·m^1/2^ at RT, with flexural strength of 676 MPa at 1800 °C, showing the potential performance development of C_f_/ZrB_2_-based composites^52^. In this work, the work of fracture of C-PDA coated C_f_/ZrB_2_-based composite is clearly higher than those of C_f_/ZrB_2_-based composites, also 2 orders of magnitude higher than that of ZrB_2_-based ceramics (~ 100 J/m^2^)^53^ and more 13 times than that of non-C-PDA coated C_f_/ZrB_2_-based composite (~ 754 J/m^2^)^35^. These attractive results demonstrate the great promise of using C-PDA to customize the interface between rigid fiber and brittle high-temperature ceramics.

## Conclusions

The electric field-assisted polymerization (EFAP) route was developed to improve the deposition time efficiency of polydopamine (PDA) coating and overcome the thickness limitation. The carbonized polydopamine (C-PDA) coating was applied as the interphase of carbon fiber-reinforced ZrB_2_-based composites (C_f_/ZrB_2_-based composite) to bond rigid fibers and brittle ceramics, where C-PDA coating was prepared by the pyrolysis of PDA coating. The main conclusions are summarized as follows:

1. Uniform and dense PDA coating with controlled thickness can be efficiently deposited on carbon fibers (C_f_). The thickness of PDA coating reaches the micron level (over 1800 nm) for the first time. The deposition rate of PDA coating reached 5589 nm/h, which was 3 orders of magnitude higher than that of the traditional self-polymerization process. The high deposition rate is attributed to the fact that EFAP promotes the oxidation process of dopamine (DA) and accelerates the aggregation and in-situ polymerization process of DA and its derivatives on the surface of C_f_. By adjusting the EFAP parameters (e.g. DA-concentration, current, and deposition time), the thickness of PDA coating could be conveniently designed from nano-scale to micro-scale.
2. C-PDA coating is well bonded on C_f_ with no visible cross-sticking among neighboring fibers. The thickness of C-PDA coating is quite dependent on the thickness of PDA coating and about half of PDA coating. The thickness of C-PDA coating can be designed by controlling the thickness of PDA. In addition, C-PDA presents a layered structure.
3. C-PDA coating can effectively tailor the interfacial bonding and improve the comprehensive performance of fiber-reinforced high-temperature ceramics composites. The use of C-PDA as the interphase of C_f_/ZrB_2_-based composites enables the composites to combine good load-bearing capacity, thermal stability, and extraordinary damage resistance. The flexural strength and fracture toughness were 300 ± 10 MPa and 8.34 ± 0.05 MPa·m^1/2^ at 1800 °C, respectively, and remained nearly constant compared to that at room temperature (RT). The work of fracture was 9936 ± 548 J/m^2^ at RT and 19,082 ± 3458 J/m^2^ at 1800 °C. These attractive results demonstrate that the application of PDA can be extended to fiber-reinforced high-temperature ceramics composites to improve the overall performance of the composite.

## Supplementary Information

Supplementary Figures.

## Data Availability

The datasets used and/or analysed during the current study available from Y.L. on reasonable request.
