# Supplementary material for: Ultrafast deposition of polydopamine for high-performance fiber-reinforced high-temperature ceramic composites
Source: Sci Rep. 2022 Nov 28;12:20489. doi: 10.1038/s41598-022-24971-3 (PMC9705713; doi:10.1038/s41598-022-24971-3)
Supplement: Supplementary file 1 — Supplementary Figures. [file 41598_2022_24971_MOESM1_ESM.docx]

Supporting Information

Ultrafast deposition of polydopamine for high-performance fiber-reinforced high-temperature ceramic composites

**Yingjun Liu^a,^*, Cheng Su^a^,** **Yufei Zu^a,b^, Xiaopeng Chen^a^, Jianjun Sha^a,b,^* and Jixiang Dai^a,b^**

^a^ Key Laboratory of Advanced Technology for Aerospace Vehicles of Liaoning Province, Dalian University of Technology, Dalian 116024, P.R. China;

^b^ State Key Lab. of Structural Analysis for Industrial Equipment, Dalian University of Technology, Dalian 116024, P.R. China.

*Email: 634144110@qq.com (Yingjun Liu); jjsha@dlut.edu.cn (Jianjun Sha).

Tel./Fax.: +86-411-84709004 (Jianjun Sha).


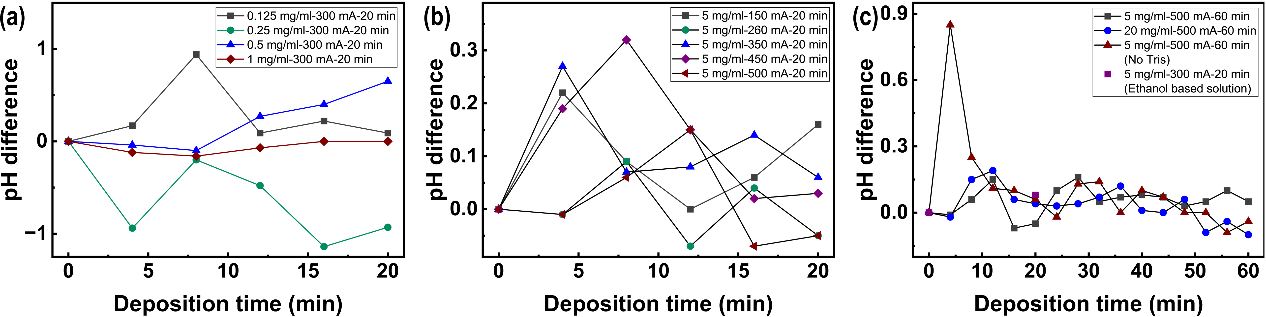


**Fig. 1S** pH difference near anode fiber and near cathode graphite plate during the electric field assisted polymerization (EFAP) process deposited at: (a) different DA-concentration, (b) different current, (c) high current, high DA-concentration with and without Tris in the water-based solution. Deposition was also performed in an ethanol-based solution. EFAP parameters have been marked. For example, EFAP parameter for a DA concentration of 5 mg/ml, a current of 500 mA, and a deposition time of 60 min are labeled as 5 mg/ml-500 mA-60 min.


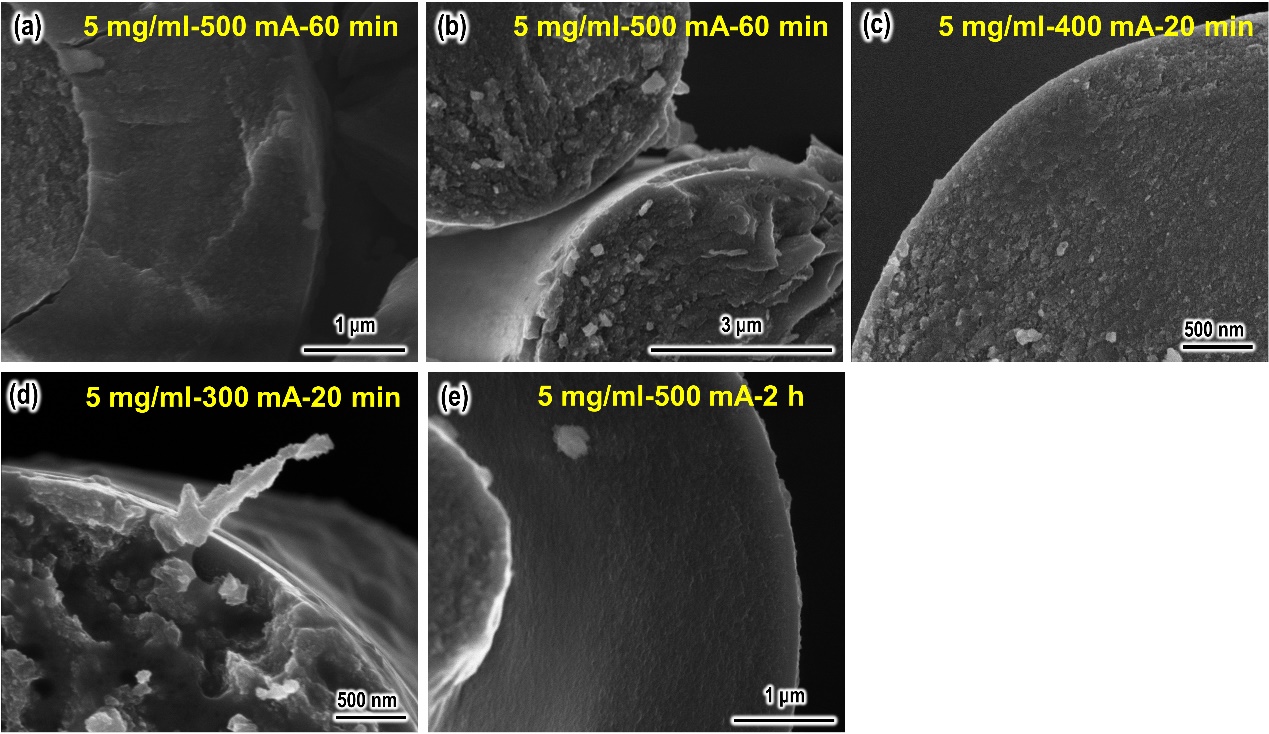


**Fig. 2S** SEM images of PDA coating deposited on fiber at: the anode (a) with and (b) without Tris in a water-based solution, (c) the cathode in a water-based solution, (d) the anode in an ethanol-based, (e) the anode in a water-based solution with a long deposition time. Deposition parameters have been marked. For example, deposition parameter for a DA concentration of 5 mg/ml, a current of 500 mA, and a deposition time of 60 min are labeled as 5 mg/ml-500 mA-60 min.

**
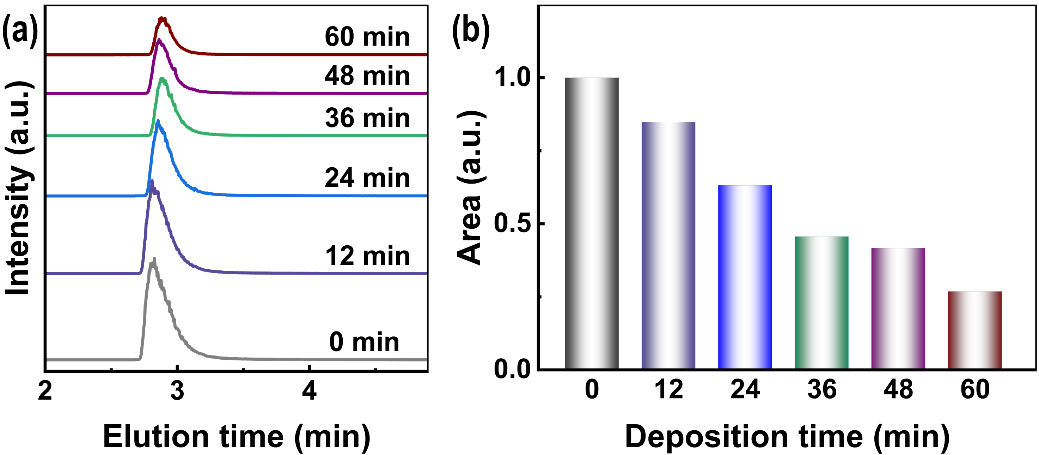
**

**Fig. 3S** (a) Chromatograms of dopamine (DA) from electric field assisted polymerization (EFAP) samples deposited at different times (with a DA-concentration of 20 mg/ml and a current of 500 mA), (b) the area from chromatographic peak gradually decreases showing the consumption of DA. The different deposition times have been marked.


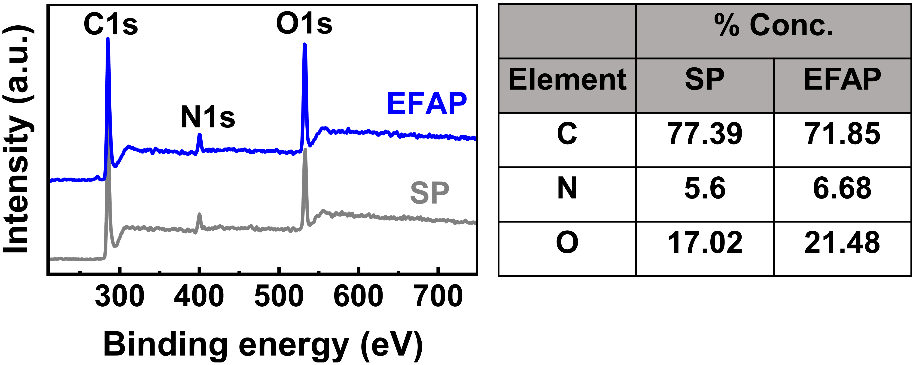


**Fig. 4S** XPS survey spectra for PDA coating prepared by self-polymerization (SP, 24 h) and electric field assisted polymerization (EFAP, with a DA-solution concentration of 5 mg/ml, a current of 400 mA and a deposition time of 20 min).

After the electric field-assisted polymerization (EFAP, with a DA-solution concentration of 5 mg/ml, a current of 400 mA and a deposition time of 20 min), the liquid sample was extracted for mass analysis. The liquid sample from traditional 24 h self-polymerization was also analyzed and used as a reference. Based on mass analysis, some oligomers were detected, as shown in Fig. 5S-Fig. 9S. For simplicity, the compound formed by the linear polymerization of DA (compounds **1**) and its oxidized derivatives (compounds **2**, **3**, **4**, **6**) was labeled as "compounds code-compounds code". For example, the following compound was labeled as **1**-**2**-**3**-**4**-**6**.


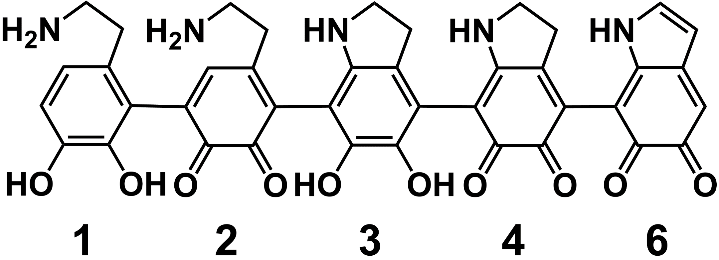


Noting that although compounds **4** and **5** share the same molecular formula (C_8_H_7_NO_2_), tending to be compounds **4** via isotopic identification^1^.


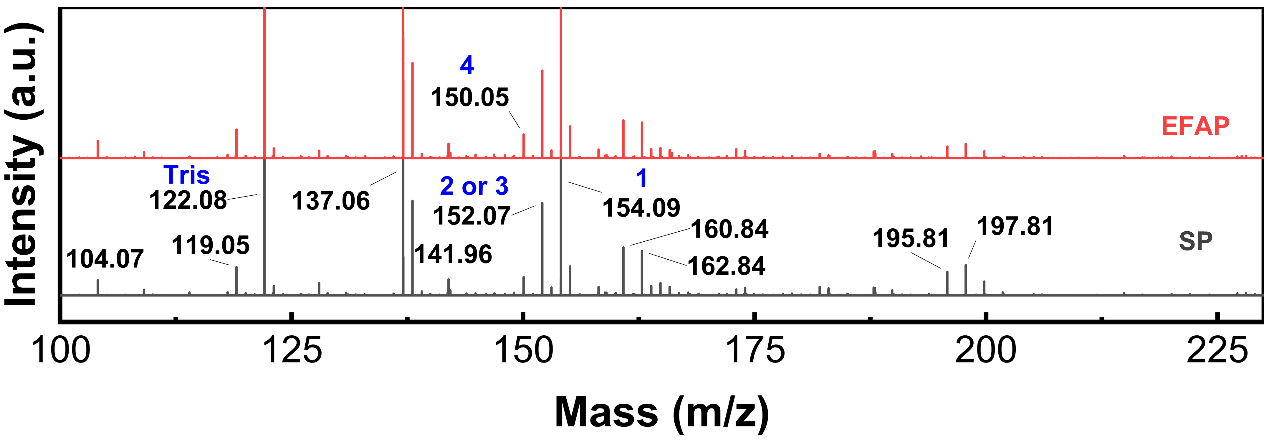


**Fig. 5S** Mass analysis on the solutions from EFAP and SP, showing monomeric compounds.


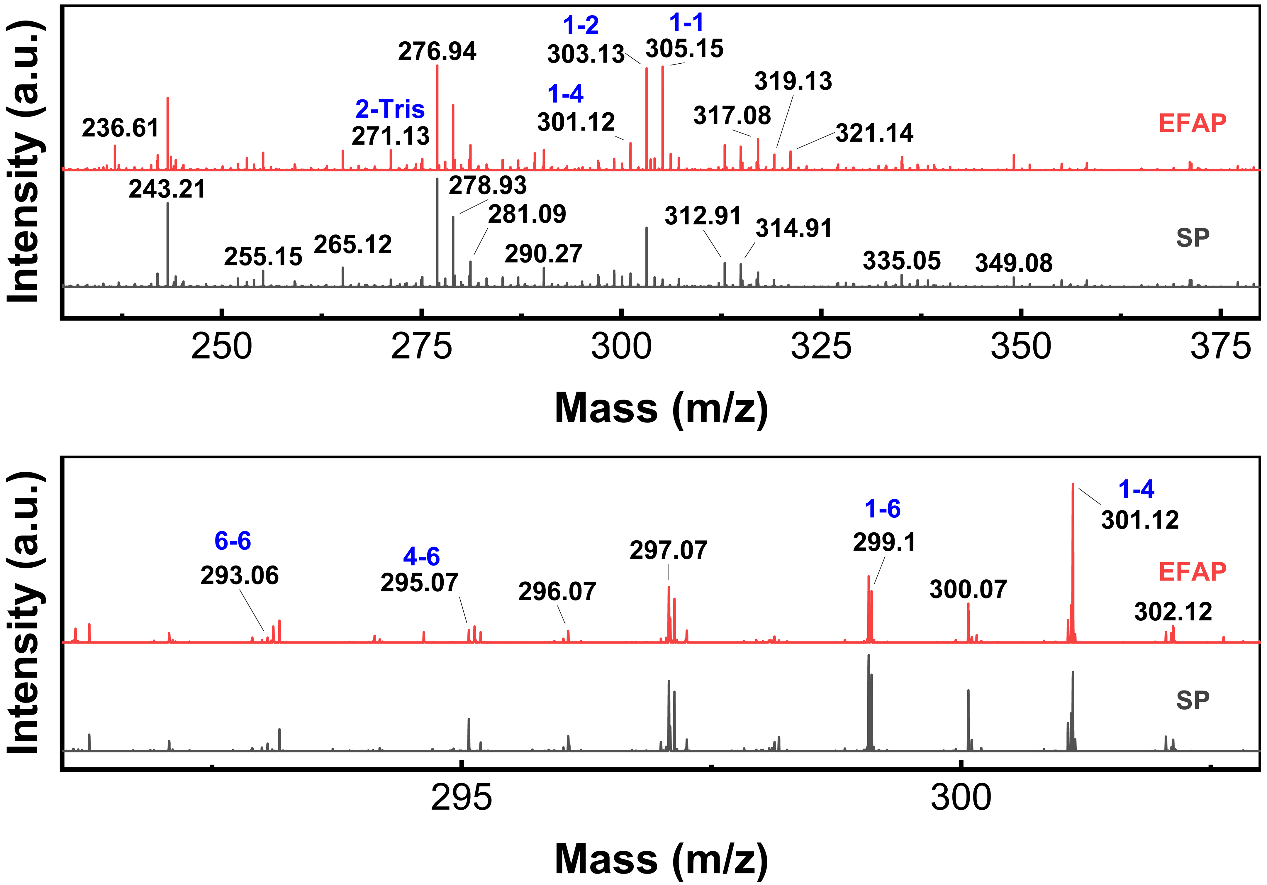


**Fig. 6S** Mass analysis on the solutions from EFAP and SP, showing dimeric compounds. Where compounds **2** may also be compounds **3**.


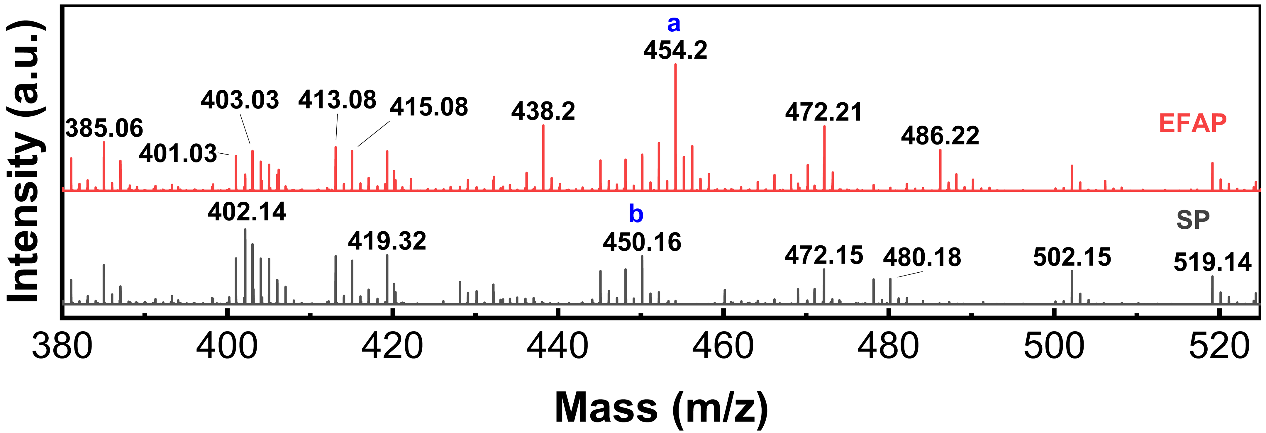


**Fig. 7S** Mass analysis on the solutions from EFAP and SP, showing trimeric compounds. Where **a** = **1**-**1**-**6** or **1**-**2**-**4**, **b** = **1**-**1**-**2**, and compounds **2** may also be compounds **3**.


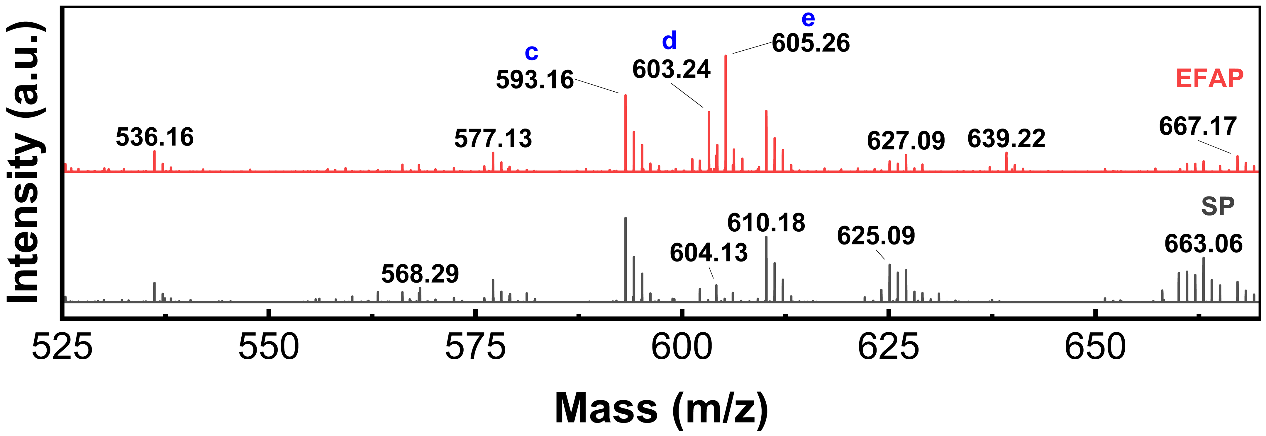


**Fig. 8S** Mass analysis on the solutions from EFAP and SP, showing tetrameric compounds. Where **c** = **1**-**2**-**6**-**6** or **1**-**4**-**4**-**6** or **2**-**2**-**4**-**6** or **2**-**4**-**4**-**4**, **d** = **1**-**1**-**1**-**4** or **1**-**1**-**2**-**2**, **e** = **1**-**1**-**1**-**2**, and compounds **2** may also be compounds **3**.


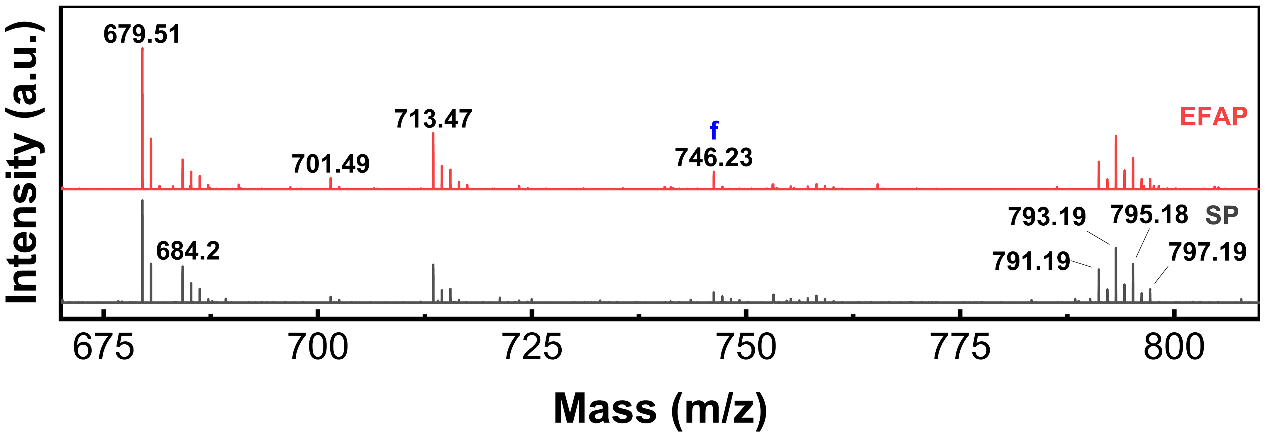


**Fig. 9S** Mass analysis on the solutions from EFAP and SP, showing pentameric compounds. Where **f** = **1**-**1**-**1**-**6**-**6** or **1**-**1**-**2**-**4**-**6** or **1**-**1**-**4**-**4**-**4** or **1**-**2**-**2**-**2**-**6** or **1**-**2**-**2**-**4**-**4**, and compounds **2** may also be compounds **3**.


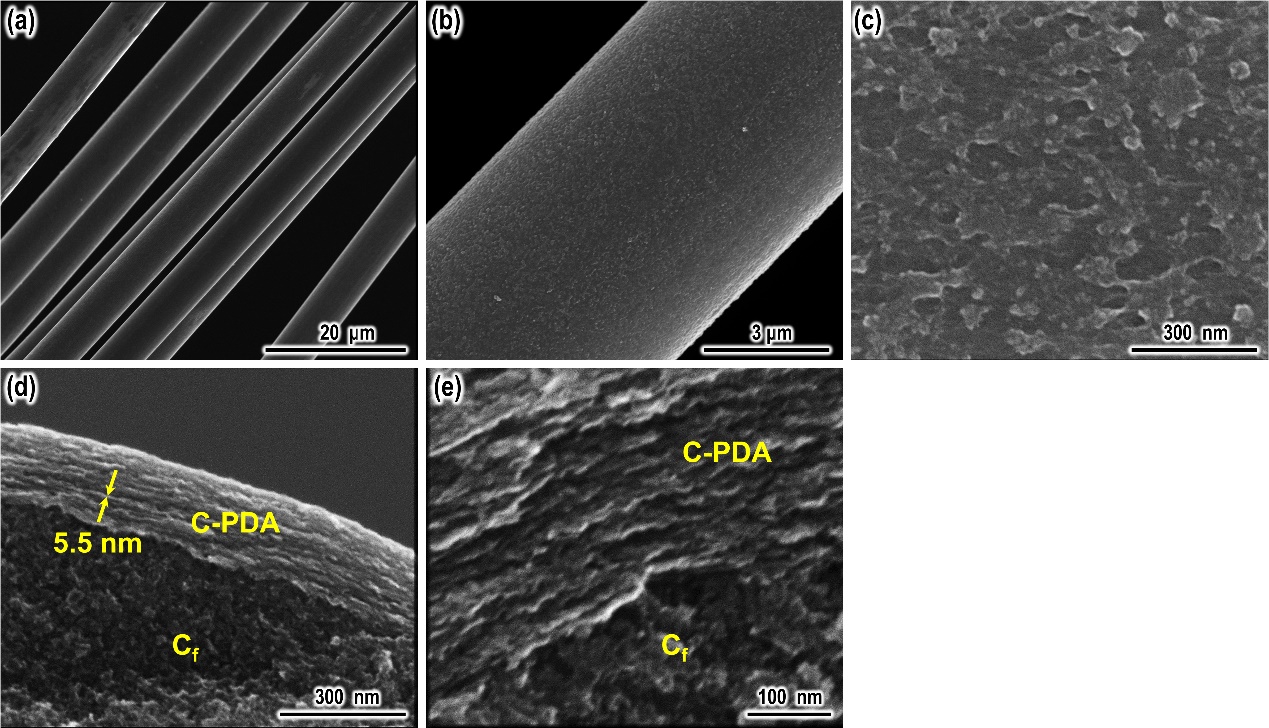


**Fig. 10S** SEM images for C-PDA coatings along (a-c) the fiber direction and (d, e) the cross-section.


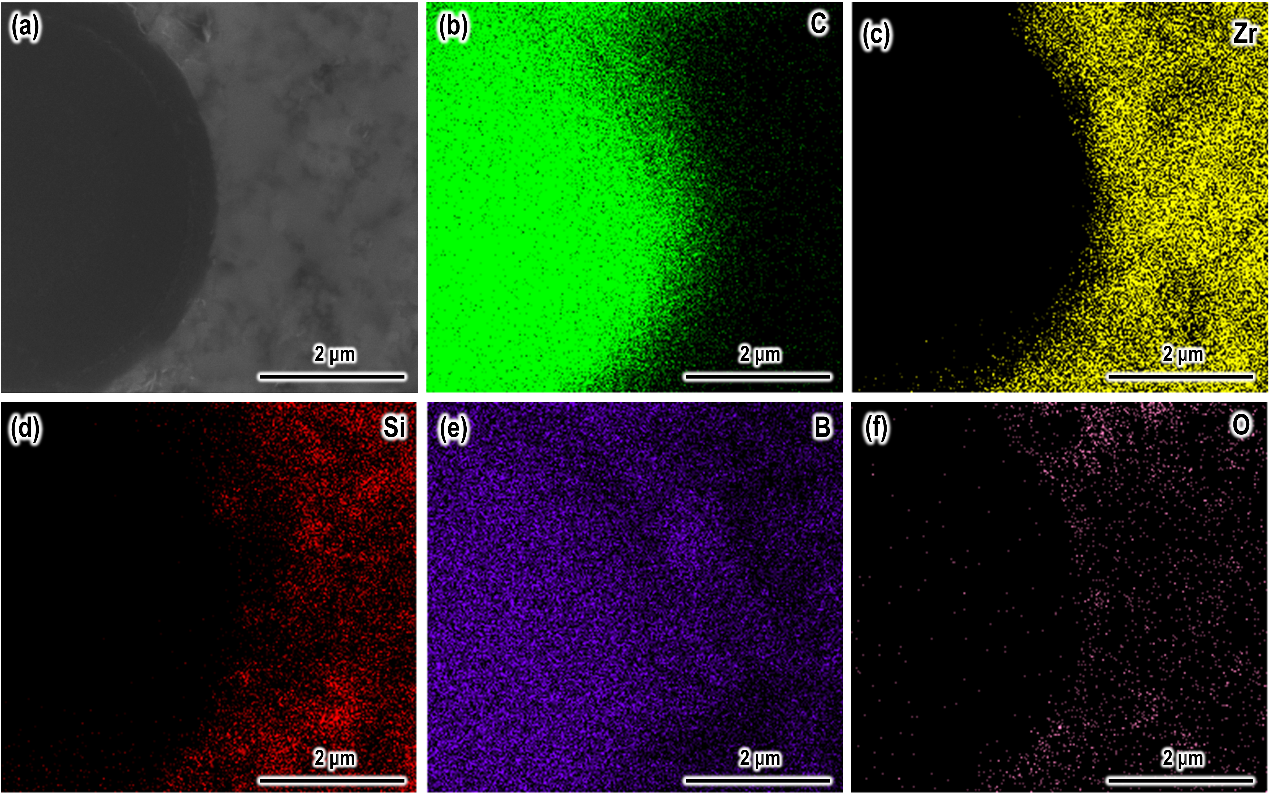


**Fig. 11S** (a-f) EDS mapping for fiber-matrix interface region of as-sintered composite. Elements have been marked.


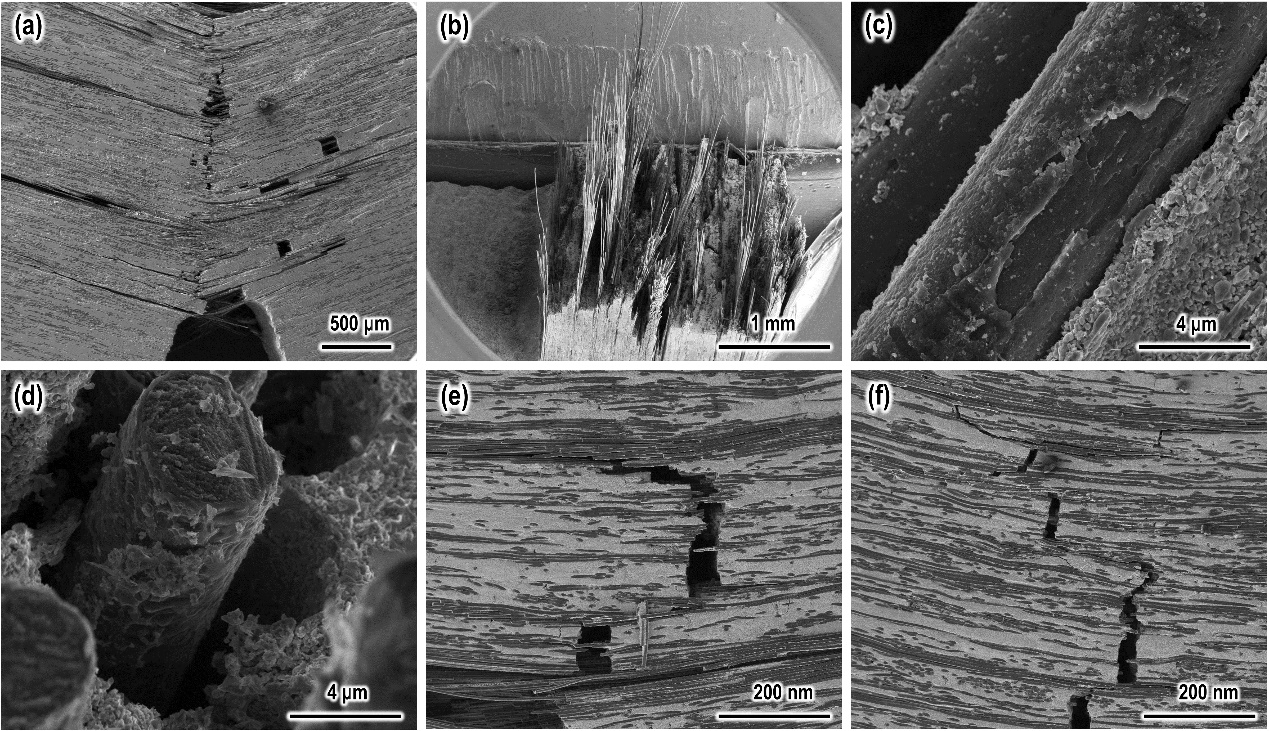


**Fig. 12S** Morphologies of the composite after SENB test at RT: (a) tortuous crack propagation path, (b) fractured surface showing extensive fiber pull-out, (c, d) C-PDA lamella peeling off. (e, f) Morphologies of the composite after SENB test at 1800 °C showing tortuous crack propagation path.

# References

1. Chan, W., Investigation of the chemical structure and formation mechanism of polydopamine from self-assembly of dopamine by liquid chromatography/mass spectrometry coupled with isotope-labelling techniques. *Rapid Commun. Mass Spectrom.* **33**, 429-436 (2019).
